# The effect of social media campaign on parental knowledge, attitudes and practices regarding the use of children car seats in the Gulf region

**DOI:** 10.1186/s12889-023-16742-0

**Published:** 2023-09-19

**Authors:** Eman A. Bakhurji, Albandari M. Alqahtani, Ezdehar M. Alwashmi, Manayer S. Husain, Balgis O. Gaffar

**Affiliations:** 1 Department of Preventive Dental Sciences, College of Dentistry, Imam Abdulrahman Bin Faisal University, 34212 Dammam, Saudi Arabia; 2 College of Dentistry, Imam Abdulrahman Bin Faisal University, 31441 Dammam, Saudi Arabia

**Keywords:** Motor vehicle crash, Car safety seat, Social media campaign, Parent knowledge, Parent awareness, Gulf Cooperation Council

## Abstract

**Background:**

Motor vehicle crashes (MVCs) are the leading cause of child deaths in the Gulf Cooperation Council. This study aimed to investigate the effect of a social media educational campaign on parents' knowledge of child safety seats.

**Methods:**

We conducted a pre-post interventional study as an online educational module in Arabic and English. The module link was shared on social media and was accompanied by a pre-post survey that included questions about demographics, knowledge, and practices of car seat use.

**Results:**

A total of 303 participants completed the campaign, with 23.8% fathers and 76.2% mothers answered the survey. The majority of participants were from Saudi Arabia (95.7%), while 4.3% were from other Gulf Cooperation Council (GCC) countries. Most parents agreed on the importance of organizing awareness campaigns and having a law to enforce the use of car seats. The pre-survey mean knowledge score was 11.64, which significantly increased to 13.1 in the post-survey (*p* < 0.001).

**Conclusions:**

The intervention of the educational campaign through social media resulted in a significant increase in parents' knowledge and awareness of the importance of using car seats correctly. This study highlights the potential effectiveness of social media campaigns in improving parents' knowledge and awareness of child safety seats.

## Background

Every year, over 1.3 million people are killed in car accidents globally [1] and around 186,300 children under 18 die annually in motor vehicle crash (MVC) [2]. MVC is the leading cause of unnatural deaths in children worldwide and a significant burden on the global economy [2]. In the United States, approximately 3% of all emergency department visits are due to MVC leading to deaths of children under the age of one [3]. Although the burden is decreasing in developed countries, MVC deaths are increasing in developing countries [1].

In comparison to Western nations, the Gulf Cooperation Council (GCC) region has significantly higher MVC and fatality rates. The recorded road traffic fatality rate in 15 developed European nations has decreased from 13.5 deaths per 100,000 populations in the 1980s to 5.5 mortality rates today. In 2013, Saudi Arabia and Oman had the highest number of full-time equivalents (FTEs) in the GCC region, followed by Kuwait, Qatar, the United Arab Emirates (UAE), and Bahrain. In terms of population size and vehicle ownership, the United Kingdom had the lowest fatality rates [4].

Despite being a developed, high-income country, Saudi Arabia has a high burden of traffic injuries [5]. According to the Global Burden of Disease (GBD), two types of injuries are among the top ten leading causes of death in Saudi Arabia: transport injuries and unintentional injuries [6]. MVC was the leading cause of injury and mortality in Saudi children, accounting for 60.6% of all cases; they were most common in 13- to 18-year-olds [7]. It kills 31% of infants aged 29 days to 5 years and 58% of children aged 6 to 12 years [7].

Aside from the risk of death, nonfatal injuries in children can result in permanent disabilities that have a long-term impact on the population's health and healthcare utilization. According to a World Health Organization (WHO) report, MVC is expected to be the leading cause of disability-adjusted life years (DALYs) loss worldwide by 2030 [8]. According to some studies, up to 70% of those injured in MVC sustain head injuries whereas facial injuries accounted for up to 11% [9]. When compared to other parts of the body, head injuries are associated with a higher risk of morbidity and mortality in adults [10, 11]. Because of the immaturity of their skeletal system and the larger head-to-body ratio, the impact of MVC injuries is magnified in children [12]. Furthermore, head injuries place a significant strain on healthcare facilities. Every year, approximately half a million children in the United States suffer from a traumatic brain injury, while in Saudi Arabia, brain injuries account for approximately 16.6% of DALYs, with MVC accounting for 8.1% of them [13, 14].

It is widely recognized that MVC injuries are preventable. Increasing passenger safety technology, child safety regulations, and safety measures have reduced infants and children deaths [15]. There is a 50%–75% reduction in child death rates when children are properly restrained in car seats and restraints that are sized for their age, weight, height, and other physical limitations (World Health Organization (WHO)) [16–20]. According to the American Academy of Pediatrics, infants and toddlers up to two years of age must sit in a rear-facing seat. For children older than two years, a forward-facing seat with a harness should be used until the child reaches 29 kg. When a child exceeds the forward-facing weight limit, the belt-positioning booster seat is used until the vehicle seat belt properly fits, which is around 8–12 years old. All children younger than 13 years old should sit in the back seat [21].

Investments in primary prevention aimed at reducing the burden of MVC lag behind in the GCC [22]. A hospital-based educational program was conducted in China to increase birthing mothers' knowledge and use of child safety restraints. The program resulted in more than 90%of mothers benefitted from the educational intervention leading to 20% increase in purchase of child safety seat (CSS) for their babies [23]. Another study found that combining a public health education program with financial incentives to increase the use of vehicle restraints can be efficacious [24]. According to the findings of previous studies, parents of young children favour the use of digital resources due to their convenience and novelty. They preferred using apps, but they seem to retain more information from reading information booklets [25].

These results indicated that car seat education programs helped parents learn more about how to use car seats correctly, choose the right type of seat, understand current rules and laws and what they need to do to ensure the child’s safety in case of a crash [26]. Therefore, the aim of this study is to assess the efficacy of a social media campaign on parents' knowledge and use of car seat safety.

## Methods

### Ethical considerations

This study was approved by the Institutional review board Deanship of Scientific Research at Imam Abdulrahman Bin Faisal University (IRB-2023–02-095). All experiments were performed in accordance with guidelines and regulations of the Declaration of Helsinki. Informed consent was obtained from all parents who participated in the study.

### Study design and setting

This study was an interventional pre–post survey conducted to test the effectiveness of an online module in improving parents’ awareness and knowledge regarding car seats use. Face and contents validity and reliability were assessed during a pilot test prior to the conduct of the study. The pilot test was conducted on a group of 30 participants and contents were modified based on its results.

### Study participants

The study targeted parents in the Gulf Cooperation Council (GCC) who have children aged from birth to 12 years old. No exclusion criteria were applied. This study used a snow-ball sampling method where current participants invite other potential participants. The recruitment began with posting the link of an online module on Twitter, Telegram, SnapChat and spreading it on WhatsApp groups.

### Sample size

The sample size was calculated using an online calculator (https://www.calculator.net/sample-size-calculator.html). Based on a previous study, [27] we determined that a sample size of 200 would be appropriate to achieve our objectives of Power is 80%, CI is 95% and alpha is 5%, with an adjustment to 300 to account for potential incomplete data and response error.

### Project preparation and data collection

Online modules were developed by the research team based on a previous study [15] and expert opinions. The online module was developed by SurveySparrow (SurveySparrow Inc, Palo Alto, CA) and the campaign logo was made with the Procreate® App. The module was made in English and Arabic versions. It consisted of three sections:

The pre-survey, Sect. 1, included a) nine Demographic questions: such as gender, country of residence, educational level, income, number of children and the children’s health status (healthy versus special needs), b) three attitude questions regarding car seat useutilizing 5-point Likert scale ranging from strongly agree with strongly disagree, c) three opinion questions towards car seat safety using the 5-point Likert scale, and d) nine pre-testing knowledge questions with multiple choice answers that included questions about type, position and age of each car seat.

The intervention, Sect. 2, Educational material, which included: numerous videos describing the significance of using a car seat ( http://www.youtube.com). the options available to parents of both normally developing and disabled children (https://youtu.be/GyDLCMHmDuk) (https://youtu.be/cj_b5nWQOD8), as well as important usage recommendations (https://youtu.be/OjbYkladmgs) This section included also photographs of each type of car seat with short and concise pieces of information. Furthermore, the WHO findings on the use of car seats in reducing child mortality, as well as international guidelines recommendations on child safety seats, were presented via multiple short statements through the module. The educational videos were done using Keynote (Apple Inc.), iMovie (Apple Inc.), and YouTube™.

After completing Sect. 2 and transferring to Sect. 3, the participants were not allowed to go back to Sect. 2 in order to ensure that their pre-testing knowledge scores is not influenced by the educational section.

The post-survey, Sect. 3, included nine post-testing knowledge questions which are the same pre-testing knowledge questions; and two feedback questions about participants opinion of the campaign which included: 1) their willingness to use children car seats after the campaign and 2) if the campaign was useful.

Each section had to be completed before moving to the next one, and participants were not allowed to go back and redo any of the sections if they had already finished it and moved to the following one. However, participants were allowed to close the module and come back to it at their convenience without time restrictions. Parents of children with special needs were directed to answer questions regarding special needs car seats while parents of healthy children were directed to answer questions for car seats recommended for healthy children. Additionally, parents were directed the educational material appropriate for their child’s need (healthy versus special needs). Those who were not parents or parents who did not have children in the specified age group were directed to exist the module after asking an eligibility question. Every person's right to privacy is upheld. The users' personal information was not collected. Immediately after finishing the survey, each person was given a different IP address.

### Statistical analysis

The data was collected and coded in an excel sheet. The COUNTIF Formula was used in Excel to check for entry duplication based on IP address. Then the data was exported to SPSS (SPSS PC, Version 22.0. Armonk, NY: IBM Corp.) for statistical analysis. Numbers and percentages were calculated for demographical and descriptive variables. Knowledge questions were given a score of one for correct answers and a score of zero for incorrect answers. Total knowledge score was calculated by summing the scores of all knowledge questions for each participant. These scores were calculated for pre and post knowledge questions for each participant. Paired t test was used to compare pre and post changes in parental knowledge scores. A *P* value of ≤ 0.05 was considered significant.

## Results

### Reliability test

The Cronbach’s alpha value (internal consistency) of the survey demonstrated acceptable reliable results of > 0.6.

### The demographics

303 of participants completed the campaign, with majority (76.2%) were mothers and (95.7%) were from Saudi Arabia. Almost half (40.6%) of the participants had more than four children, while majority of them had healthy children (92.4%). Most parents had a university educational level or above (72%) and (66.7%) for mothers and fathers, respectively. About half of the participating parents (46.5%) had an income of more than 10,000 Saudi Riyals, (Table 1).

**Table 1:** Background information of the study participants (*N* = 303)

Demographics		Frequency n (%)
Gender	Father	72 (23.8)
	Mother	231 (76.2)
Country of Residence	Saudi Arabia	290 (95.7)
	Other countries	13 (4.3)
Maternal Education	No education or School education	85 (28)
	University and above	218 (72)
Paternal Education	No education or School education	101 (33.3)
	University and above	202 (66.7)
Family Income (SAR)	< 4000 SAR	36 (11.9)
	> 4000–7000 SAR	44 (14.5)
	> 7000–10000 SAR	82 (27.1)
	> 10,000 SAR	141 (46.5)
Number of Children	One child	62 (20.5)
	Two children	58 (19.1)
	Three children	60 (19.8)
	Four and more children	123 (40.6)
Children’s Medical History	Healthy child/children only	280 (92.4)
	Special needs child/ Children only	5 (1.7)
	Both (healthy and special needs children)	18 (5.9)

### Attitude and practices regarding car seats use

**Fig. 1:**
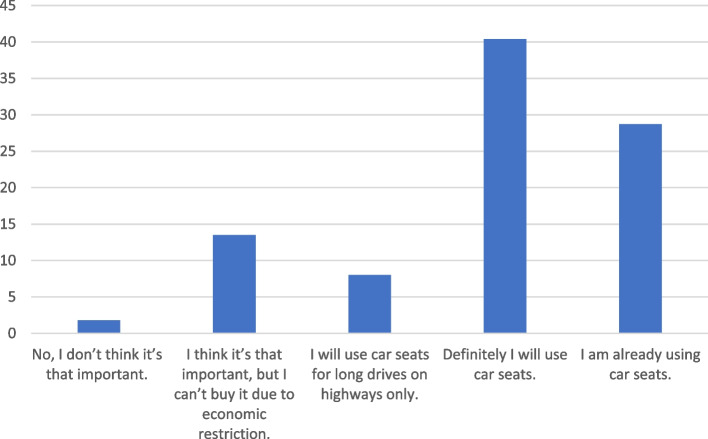
Willingness to use the car seats after campaign education

Table 2 represents participants practices regarding car seats use, with 88.8% of parents agreed that car seats should be used for all children and agreed that their babies shouldn’t sit in their laps. On the contrary,76.2% disagreed that their children should sit in the back without car seats. However, 70% of parents agreed that there is a lack of awareness about the importance of car seats in their countries. The majority of parents agreed on the importance of organizing awareness campaigns (89.4%) and having a law to enforce the use of car seats (81.5%). Figure 1 shows the willingness of parents to use the safety seat after the campaign. Forty percent of participants indicated that they would use safety seats.

**Table 2:** Attitude towards car seat safety

	**Practice ** ***N *= 303**	**Frequency n (%)**
Car seats must be used for all children	Strongly disagree/ Disagree	19 (6.3)
	Neutral/ I don’t know	15 (5)
	Strongly agree/ Agree	269 (88.8)
Babies should be seated on their parents lap	Strongly disagree/ Disagree	269 (88.8)
	Neutral/ I don’t know	16 (5.3)
	Strongly agree/ Agree	18 (5.9)
Children can be seated in the back seat without car seat	Strongly disagree/ Disagree	231 (76.2)
	Neutral/ I don’t know	54 (17.8)
	Strongly agree/ Agree	18 (5.9)
Parents in my country lack awareness of the importance of car seats	Strongly disagree/ Disagree	39 (12.9)
	Neutral/ I don’t know	51 (16.8)
	Strongly agree/ Agree	213 (70.3)
The competent authorities should organize awareness campaigns to educate and train parents on child car seats	Strongly disagree/ Disagree	7 (2.3)
	Neutral/ I don’t know	25 (8.3)
	Strongly agree/ Agree	271 (89.4)
I’m with the law that enforce the use of the children car seats	Strongly disagree/ Disagree	30 (9.9)
	Neutral/ I don’t know	26 (8.6)
	Strongly agree/ Agree	247 (81.5)

### Parental knowledge before and after the campaign

Table 3 shows item and overall knowledge scores before and after the educational material. The overall mean knowledge score at baseline was 11.64 ± 1.73) which was significantly improved after the educational content to 13.1 ± 1.69 in the post-survey (*p* = 0.000). The highest item analysis score was knowledge about the correct position safety seat in the car (1.86 ± 0.34 Vs 1.85 ± 0.35; *p* = 0.687). While the lowest score was regarding the criteria of choosing the appropriate safety seat (0.11 ± 0.41 Vs 0.13 ± 0.48; *p* = 0.000).

**Table 3:** Item analysis of knowledge questions before and after the educational material

Question	Mean ± std. deviation		sig
	Pre	Post	
Q1: Which of the following car seats is suitable for 6 to 11 years old children?	1.37 ± 0.48	1.53 ± 0.5	< 0.001
Q2: Which of the following car seats is suitable for 4 to 6 years old children?	1.48 ± 0.5	1.5 ± 0.5	0.670
Q3: Which of the following car seats is suitable for 1 to 4 years old children?	1.42 ± 0.49	1.33 ± 0.47	< 0.001
Q4: At what age children should sit in rear facing car seats?	1.25 ± 0.43	1.46 ± 0.50	0.047
Q5: what is/ are the criteria for choosing the right car seats?	1.60 ± 0.49	1.83 ± 0.38	< 0.001
Q6: what is/ are the criteria for choosing the right car seats for special health care needs?	0.11 ± 0.41	0.13 ± 0.48	< 0.058
Q7: As a result of using appropriate car seats and a restrain, what is the approximate decrease in fatality rate?	1.32 ± 0.47	1.72 ± 0.45	< 0.001
Q8: where should the car seat be positioned in the car?	1.86 ± 0.34	1.85 ± 0.35	0.687
Q9: until what age does the child sit in designated car seats?	1.24 ± 0.42	1.66 ± 0.47	< 0.001
Total score	11.64 ± 1.73	13.1 ± 1.69	< 0.001

### Perceptions/ satisfaction on the campaign

**Fig. 2:**
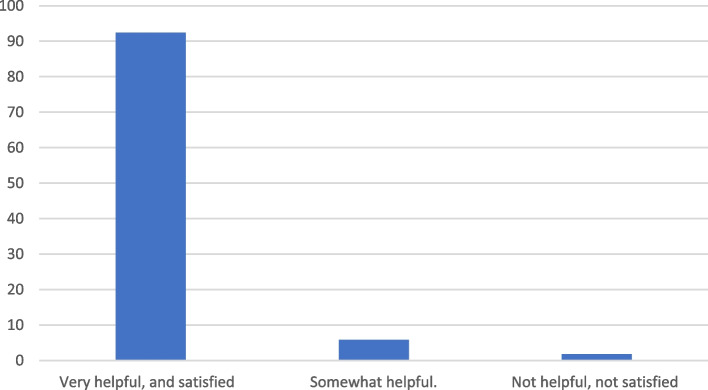
Participants perceptions regarding the campaign

Participants were asked to share their perceptions regarding the campaign, and most of them (92.4%) thought that it was very helpful and they were satisfied with the contents provided (Fig. 2).

## Discussion

The goal of the present study was to measure the effectiveness of a social media educational campaign on parental knowledge and attitudes regarding safety car seats. The social media educational campaign of the present study was well perceived by parents and resulted in an improvement in parental knowledge and attitude towards safety car seats. It offers evidence that educational campaigns spread across social media can increase total knowledge significantly between the pre and post-survey results. There was also a significant increase in all nine knowledge question areas included in the assessment.

In spite of the increased enforcement of traffic laws in Kingdom of Saudi Arabia (KSA), motor vehicle crashes (MVC) continue to be a major cause of disabilities and death in the community [2, 5, 28–30]. Saudi law requires parents to use safety equipment like car seats and seatbelts for their children. However, only 15.3% of drivers and passengers regularly use seatbelts [31]. In a study conducted in KSA, approximately 30% of children under 5 were restrained, with the most common method being in the front seat and on an adult passenger's lap (54.5%). Another KSA study reported that 53.8% of the children were found to be riding without a seatbelt or safety seat in the back seat [17]. On the contrary, more than 90% of people using car seats and safety restraint systems in the United States and Australia [2].

The use of appropriate child car seats plays an important role in reducing children's mortality due to car accidents; however, a study conducted in Romania in 2020 reported that the majority of drivers were aware of the existence of child safety restraint regulations and are using car seats for their children (68.4%), but they had less knowledge when asked about the appropriate age for car seats. Those who did not use car seats in the Romanian study, listed having less knowledge, and financial reasons as reasons for not using car seats [32]. Another study done in China showed low number of parents who owned car seats (27.8%) while only 22.9% of them were using car seats, especially those who had high knowledge towards safety measures [33]. In Unaizah, KSA, most of the parents (64.3%) did’nt use car seats and their knowledge regarding their importance was considered low [15]. Therefore, awareness of parents in KSA needs to be raised in order to keep their children safe while they are out on the road. Indeed, the current research provides a program that was effective in boosting awareness among parents whom, in general, do not get this kind of campaign. It is hoped that this would lead to a reduction in the wide discrepancy that exists in the rates of car seat use.

This research used a variety of learning tools to boost knowledge, including videos, messages, and quizzes spread through social media platforms to reach large number of parents of different backgrounds. Social media is an effective tool that can be used to deliver information and spread knowledge. In recent years, it became a new platform for health promotion in many areas including smoking cessation, exercising, and diet [34]. News-media campaigns that were conducted through radio, television, newspapers, and internet have effectively motivated behaviours that reduced sport-related injuries and injuries from car accidents [34]. In addition to having a large-scale audience, social media allows sharing experience and interaction between users which makes it more influential than traditional campaigns [34, 35]. Because of the wide gap in car seat use rates, the current research was carried out through an internet campaign in the hopes of narrowing that gap. Evidence suggests the effectiveness of combined interventions on child safety seats use. For instance, laws along with educational programs or community-wide enforcement campaigns with incentive while education-only programs were found to be less effective [36]. 

The results of this research study suggest that the majority of participants in the Arabian Gulf region were well educated mothers from Saudi Arabia. Furthermore, most participants had an income of more than 10,000 Saudi Riyals. This is consistent with other studies conducted in the region which have found similar socio-ecenomic distribution of participants [15, 27, 37]. However, this might have happened as a result of having an online campaign on social media where most its audience are from higher socio-economic background. A large specimen of the population who are from low socioeconomic background might have been excluded from benefiting from the educational program which is a drawback of this study. their exclusion might not because of their lack of access. It is mostly due to their lack of interest and willingness to participate in such programs. More attention should be given to those who do not engage in online educational campaigns by in-person programs.

The results of this study found that the overall mean knowledge score of post-test was significantly higher than pre-test. The highest item analysis score was knowledge about the correct position safety seat in the car, while the lowest score was regarding the criteria of choosing the appropriate safety seat. These findings demonstrate that social media educational campaigns can be an effective tool for increasing parental knowledge, attitude and practice of car seat use worldwide. Additionally, it can be effective in increasing awareness and knowledge about health-related topics [38, 39]. Furthermore, the high satisfaction rate reported by participants in this study (92.4%) indicates that such campaigns may be well received by parents in the Arabian Gulf region. However, further research is needed to determine if these effects are sustained over time and if they are associated with improved outcomes for children using car seats in this region or elsewhere.

### Study limitations and future directions

It is important to note that there are some limitations to this study. First, this study relied on self-reported practices, which may not reflect actual behaviours and may lead to questioning the reliability of the answers as over-reporting is likely to occur. Such design is subjected to several types of bias, such as self-selection bias and social desirability bias. However, it remains among the best available tools to assess the population's knowledge, attitude, and reported practices. Additionally, the immediate assessment of improvement in knowledge may not necessarily mean maintaining long-term knowledge. Furthermore, an improvement in knowledge does not guarantee a change in behaviour, as most of our participants knew the importance of car seats, but there was little compliance from parents. The study included parents who had internet access, which may not reflect the situation of those without internet access, which could lead to selection bias. However, our study had participants from low socio-economic status, which indicates inclusivity of different backgrounds. Future research should look at the effectiveness of social media educational campaigns over a period of time to see whether they have sustained long-term impact. This might be accomplished by conducting pre-post questionnaires over a time lag (e.g., 6 months or a year between). Additionally, consider larger sample sizes and longer time frames to better understand the impact of social media. Furthermore, monitoring parents' capacity to appropriately install car seats after interventions might be useful in developing future programs.

## Conclusions

This study provides evidence that implementing social media educational campaigns can be an effective tool for increasing parental knowledge, attitude, and practice of car seat use in the Arabian Gulf region. The high satisfaction rate reported by participants suggests that such campaigns may be well received by parents in this region. The use of social media campaigns might help overcome some of the limitations of prior studies, such as lack of time and resources for reaching wide audience. Future research should consider larger sample sizes and longer time frames to better understand the impact of such campaigns on parental knowledge, attitude, and practice of car seat use in the Arabian Gulf region.

## Data Availability

The datasets used and/or analysed during the current study are available from the corresponding author on reasonable request.

## Abbreviations

MVC: Motor Vehicle Crash
FTEs: Full-Time Equivalents
GCC: Gulf Cooperation Council
DALYs: Disability-Adjusted Life Years
GBD: Global Burden of Disease
WHO: World Health Organization
